# CRISPR-Cas12a System With Synergistic Phage Recombination Proteins for Multiplex Precision Editing in Human Cells

**DOI:** 10.3389/fcell.2021.719705

**Published:** 2022-06-14

**Authors:** Chengkun Wang, Qiong Xia, Qianhe Zhang, Yuanhao Qu, Stephen Su, Jason K. W. Cheng, Nicholas W. Hughes, Le Cong

**Affiliations:** ^1^ Department of Pathology, Stanford University School of Medicine, Stanford, CA, United States; ^2^ Department of Genetics, Stanford University School of Medicine, Stanford, CA, United States; ^3^ Wu Tsai Neuroscience Institute, Stanford University, Stanford, CA, United States

**Keywords:** CRISPR, genome editing, Cas12a, RecT, single-stranded annealing protein

## Abstract

The development of CRISPR-based gene-editing technologies has brought an unprecedented revolution in the field of genome engineering. Cas12a, a member of the Class 2 Type V CRISPR-associated endonuclease family distinct from Cas9, has been repurposed and developed into versatile gene-editing tools with distinct PAM recognition sites and multiplexed gene targeting capability. However, with current CRISPR/Cas12a technologies, it remains a challenge to perform efficient and precise genome editing of long sequences in mammalian cells. To address this limitation, we utilized phage recombination enzymes and developed an efficient CRISPR/Cas12a tool for multiplexed precision editing in mammalian cells. Through protein engineering, we were able to recruit phage recombination proteins to Cas12a to enhance its homology-directed repair efficiencies. Our phage-recombination-assisted Cas12a system achieved up to 3-fold improvements for kilobase-scale knock-ins in human cells without compromising the specificity of the enzyme. The performance of this system compares favorably against Cas9 references, the commonly used enzyme for gene-editing tasks, with improved specificity. Additionally, we demonstrated multi-target editing with similar improved activities thanks to the RNA-processing activity of the Cas12a system. This compact, multi-target editing tool has the potential to assist in understanding multi-gene interactions. In particular, it paves the way for a gene therapy method for human diseases that complements existing tools and is suitable for polygenic disorders and diseases requiring long-sequence corrections.

## Introduction

The powerful CRISPR technologies have been widely applied in various biological systems and are greatly changing the field of genome engineering (Cong et al., 2013; Jinek et al., 2013; Hsu et al., 2014; Komor et al., 2017; Knott and Doudna, 2018). Cas12a(Cpf1), a member of the Class 2 Type V CRISPR-associated endonuclease family, differs from Cas9 in several ways (Zetsche et al., 2015): it has a T-rich PAM recognition site (TTTV) while Cas9 PAM site is G-rich (NGG) (Zetsche et al., 2015; Yamano et al., 2016); its guide RNA is shorter in sequence than Cas9 (Zetsche et al., 2015); Cas12a cuts DNA distal and downstream relative to the PAM sequence and results in staggered ends while Cas9 cuts DNA proximal and upstream and results in blunt ends; and Cas12a is reported to be more precise and induces less off-target activities than Cas9 (Zetsche et al., 2015; Kim et al., 2016; Kleinstiver et al., 2016; Yamano et al., 2016; Swarts et al., 2017). Moreover, due to its intrinsic ribonuclease activity that processes its own crRNA, Cas12a is capable of multiplex editing from a single RNA transcript (Zetsche et al., 2015; Zetsche et al., 2017).

Multiplex engineering can be useful in understanding gene-gene interactions, combinatorial effects of different genetic mutations, and biological pathways with redundancy between individual genes (Tak et al., 2017; Li et al., 2019; McCarty et al., 2020). Along with its other beneficial properties, Cas12a holds great potential in serving as a tool to solve genome engineering problems. However, similar to Cas9, Cas12a relies on endogenous homology-directed repair (HDR) mechanisms to achieve precise gene modifications, but the HDR efficiency is often very limited due to the competing error-prone repair pathways which happen much more frequently (Pickar-Oliver and Gersbach, 2019; Anzalone et al., 2020). While many previous studies have been done to improve the efficiency of base editing and short genomic alterations, large-scale genome editing using Cas12a still remains a challenge (Li et al., 2018; Kleinstiver et al., 2019; Wang et al., 2020).

Previously we have identified that phage single-stranded annealing proteins (SSAPs) could enhance Cas9-based gene editing efficiencies in mammalian cells, and among all SSAPs screened, *E. coli* RecT exhibited the largest increase (Wang et al., 2021). We termed this high-efficiency Cas9 editing method as RecT Editor *via* Designer-Cas9-Initiated Targeting (REDIT). Here, combining the efficiency of REDIT and the multi-target editing ability of Cas12a, we developed a new Cas12a-REDIT method.

## Materials and Methods

### Plasmids Construction

The fragment encoding the scFV-RecT or GFP was Gibson assembled into MS2-P65-HSF1-GFP (addgene, 61423) using NEBuilder HiFi DNA Assembly Master Mix (New England BioLabs), the MS2-P65-HSF1-GFP plasmid digested with BsiWI and EcoRI. The fragment encoding Cas12a-10x GCN4 was Gibson assembled into the pX330 (42230; addgene), pX330 was digested with AgeI and EcoRI. The guide RNAs were then inserted using Golden Gate cloning (guideRNA sequence used in this study could be find in the Supplementary Table). All plasmids were sequence-verified (Eton and Genewiz) through Sanger sequencing and will be deposited to Addgene for open access.

### Cell Culture

Human Embryonic Kidney (HEK) 293T cells was ordered from ThermoFisher; Hela and HepG2 were obtained from American Type Culture Collection (ATCC). All three cell lines were maintained in Dulbecco’s Modified Eagle’s Medium (DMEM, Life Technologies), with 10% fetal bovine serum (FBS, BenchMark), 100 U/ml penicillin, and 100 μg/ml streptomycin (Life Technologies) at 37°C with 5% CO_2_.

### Transfection

HEK293T cells were seeded into 96-well plates (Corning) 12 h prior to transfection at a density of 3E4 cells/well, and 250 ng of total DNA was transfected per well using Lipofectamine 3000 (Life Technologies) following the manufacturer’s instruction. HeLa and HepG2 cells were seeded into 48-well plates (Corning) 1 day prior to transfection at a density of 5E4 and 3E4 cells/well respectively, and 400 ng of total DNA was transfected per well.

### Fluorescence-Activated Cell Sorting (FACS)

72 h after transfection, cells were washed with PBS and dissociated using 50 μl TrypLE Express Enzyme (Thermo Fisher Scientific). Cell suspension was then transferred to a 96-well U-bottom plate (Thermo Fisher Scientific) and centrifuged at 300 g for 5 min. After removing the supernatant, pelleted cells were resuspended with 50 μl 4% FBS in PBS, and the mKate or GFP knock-in efficiency was analyzed on a CytoFLEX flow cytometer (Beckman Coulter; Stanford Stem Cell FACS Core) within 30 min.

### Next-Generation Sequencing (NGS) Library Preparation

48–72 h after transfection, genomic DNA (gDNA) was extracted using QuickExtract DNA Extraction Solution (Lucigen). 200 ng total gDNA was used for NGS library preparation. Genes of interest were amplified using specific primers (Supplementary Table) for the first round of PCR reaction, and Illumina adapters and index barcodes were added in the second round of PCR. Final PCR products were run on a 2% E-gel (Thermo Fisher Scientific) and extracted using the Monarch DNA Gel Extraction Kit (New England BioLab). The purified product was quantified with Qubit dsDNA HS Assay Kit (Thermo Fisher) and loaded for sequencing using Illumina MiSeq paired-end PE300 kits following the manufacturer’s instructions.

### High-Throughput Sequencing Data Analysis

The sequencing data was analyzed using CRISPResso2 as previously described (Clement et al., 2019; Wang et al., 2021). Briefly, the sequencing reads were demultiplexed, trimmed and merged, then aligned to reference or expected HDR amplicons. Only reads perfectly matched to th expected amplicon were considered for HDR quantification. The computation work was supported by the SCG cluster hosted by the Genetics Bioinformatics Service Center (GBSC) at the Department of Genetics of Stanford. All sequencing data have been deposited by the authors into NCBI SRA archive, and could be identified by the accession ID PRJNA735023.

### Statistical Analysis

Unless otherwise stated, all statistical analysis and comparison were performed using *t*-test, with 1% false-discovery-rate (FDR) using a two-stage step-up method of Benjamini, Krieger and Yekutieli. All experiments were performed in triplicates unless otherwise noted to ensure sufficient statistical power in the analysis.

## Results and Discussion

**FIGURE 1:**
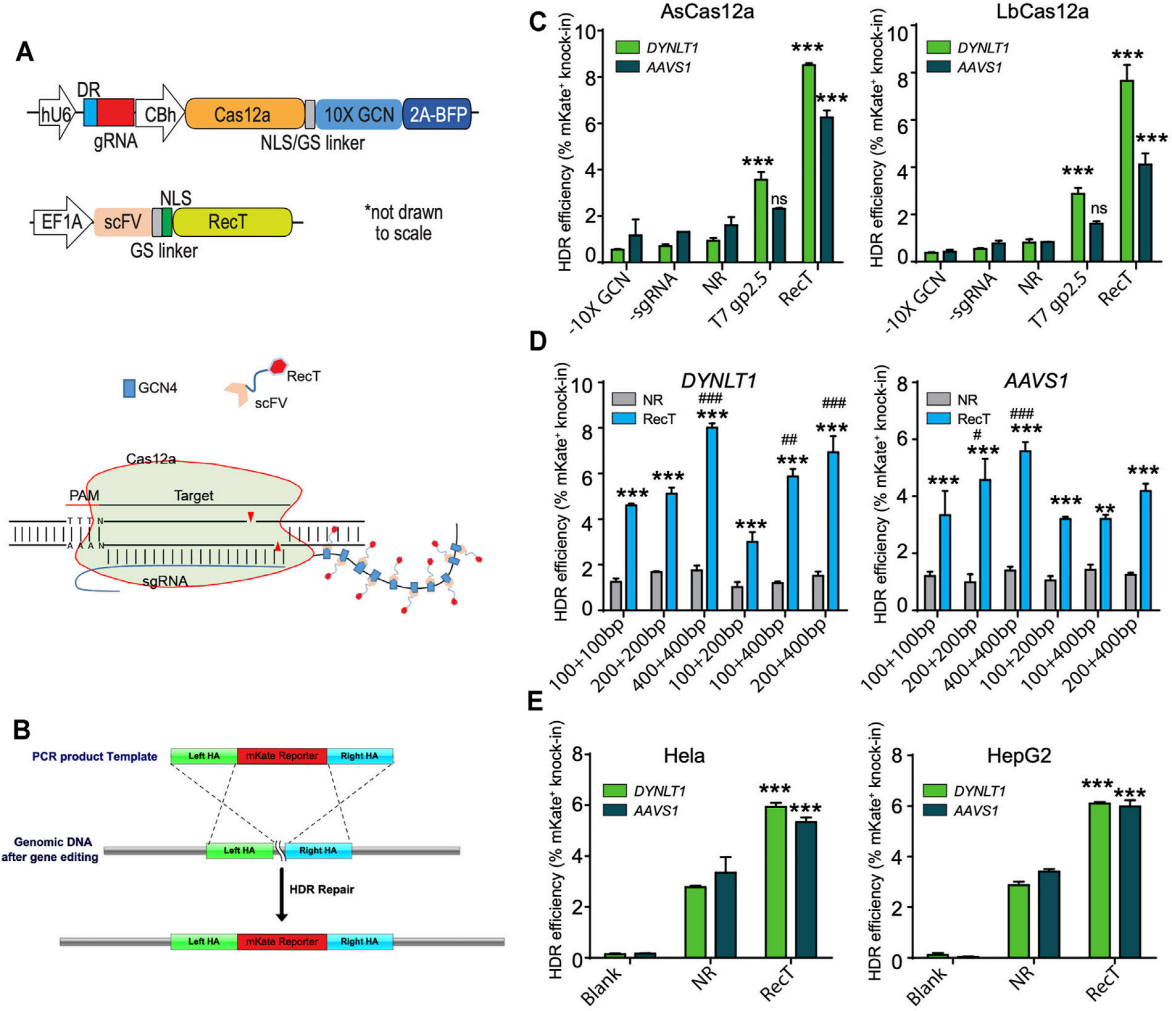
Testing Cas12a-REDIT for genome engineering in mammalian cells. **(A)** Vector designs on the top and schematic on the bottom showing the SunTag-based recruitment system of SSAP RecT and Cas12a protein. The 10X GCN4 peptide was fused expression with Cas12a through a nucleoplasmin NLS-GS peptide linker, and BFP was spliced expression by T2A peptide. The RecT was fused expression with scFV through a SV40 NLS-GS peptide linker. **(B)** Schematic showing the 2A-mKate template design and genomic knock-in assay of HDR editing. **(C)** Comparison of HDR efficiencies using different SSAPs and Cas12a homologs on *DYNLT1* and *AAVS1* sites. NR, Cas12a control without RecT (No-RecT, NR) to measure the baseline level of editing. All results here and later are from replicate experiments (*n* = 3), with error bars representing standard error of the mean (S.E.M.), unless otherwise noted. ***p < 0.001 verus NR group. ns, no significant verus NR group. **(D)** Comparison of HDR efficiencies using different template designs on *DYNLT1* and *AAVS1* sites. **p < 0.01, ***p < 0.001 verus NR group; ##*p* < 0.01, ###*p* < 0.001 verus 100 + 100bp group. **(E)** Validation of Cas12a-REDIT across different human cell types in HeLa and HepG2 cell lines. ***p < 0.001 verus NR group. SSAP, single-stranded annealing protein; NLS, nuclear localization signal; GS, glycine-serine linker; BFP, blue fluorescent protein; HDR, homology-directed repair.

We fused RecT to Cas12a *via* the previously reported SunTag-based recruitment system (Tanenbaum et al., 2014; Huang et al., 2017). In our design, Cas12a linking a tandem repeat of 10 copies of GCN4 peptides were cloned into a mammalian expression vector. The RecT protein (GFP protein in control) linking a single chain variable fragment (ScFv) of the anti-GCN4 antibody, was cloned into another vector. Upon the co-expression of two plasmids, ScFv antibodies would bind to their cognate peptide pair GCN4, recruiting RecT to the Cas12a protein (Figure 1A).

We tested our system *via* a genomic knock-in assay where a repair template of a T2A-mKate cassette (∼1 kb in length) was provided. The template bears homology arms (HA) on both sides that match the last exon (in-frame before the stop codon) of the target genes (Figure 1B). HDR efficiency was then quantified by fluorescence-activated cell sorting (FACS) where the percentage of mKate-positive cells represents successful knock-in.

Many types of Cas12a orthologs have been found across different bacteria; among those, *Acidaminococcus sp.* Cas12a (AsCas12a) and *L. bacterium* Cas12a (LbCas12a) are the two that are most efficient and commonly used (Zetsche et al., 2015). Therefore, we tested our design using these two orthologs in the HEK293T cell line. As expected, across two different gene targets (*DYNLT1* and *AASV1*), there were significant increases of HDR efficiency when RecT was recruited compared to the control in both of the Cas12a variants. Moreover, AsCas12a performed slightly better compared to LbCas12a. Hence, we further optimized our design using AsCas12a in the rest of study (Figure 1C).

As we have previously shown that the template HA length is a deciding factor of the HDR efficiency (Wang et al., 2021), we investigated such influence in Cas12a-REDIT by testing different HA designs. We found that symmetric HA performed better than asymmetric HA in general, and while our design could work with as short as ∼100 bp template HA on each side, the efficiency increased as the HA length increased. A template that had ∼400 bp HA on both sides performed the best among all six designs tested (Figure 1D).

Next, we examined our designs in two more different human cell lines, Hela (cervix-derived) and HepG2 (liver-derived), in order to evaluate the generalizability of our system. As expected, there were notable improvements of nearly 2-fold in the HDR efficiency (Figure 1E).

**FIGURE 2:**
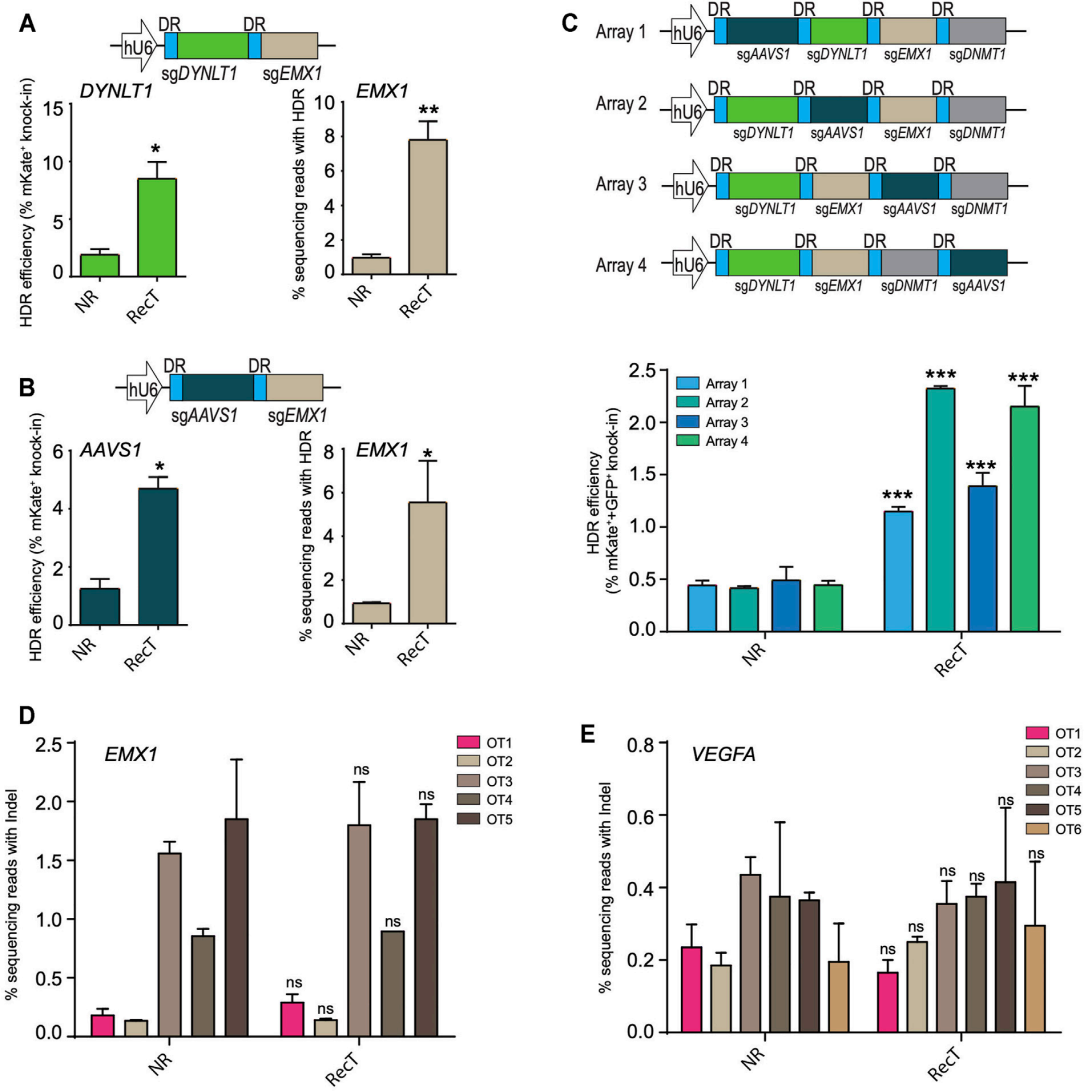
Cas12a-REDIT improves multiplexed genome editing efficiency without compromising the specificity of Cas12a. **(A**,**B)** HDR efficiencies of Cas12a and Cas12a-REDIT in the case of simultaneously targeting **(A)** *DYNLT1* and *EMX1* sites and **(B)** *AAVS1* and *EMX1* sites. Top schematic shows the vector design; mKate is inserted to *DYNLT1*/*AAVS1* locus and a 16 bp restriction site is inserted to EMX1 locus. *p < 0.1, **p < 0.01 versus NR group. **(C)** Schematic on the top showing vector designs of 4-guide RNA arrays with four guides in different order. mKate is inserted into *DYNLT1* site and GFP is inserted into *AAVS1* site, and HDR efficiencies of successful knock-in on both sites are shown on the bottom. ***p < 0.001 versus NR group. **(D**,**E)** High-throughput NGS measurement of on/off-target editing events on known off-target sites of *EMX1* and *VEGFA* locus. ns, no-significant versus NR group.

To investigate whether the improvement persisted in the case of multi-target editing, two genes *DYNLT1* and *EMX1* were targeted at the same time where mKate was inserted into *DYNLT1* and a 16bp insertion was made into *EMX1*. For the latter, next-generation sequencing was performed to assay the editing efficiency. There was a nearly four-fold increase of editing efficiency for both long and short knock-in editing when using the Cas12a REDIT method (Figure 2A). This enhancement of editing efficiencies in multiple-target settings were further validated by changing *DYNLT1* locus to *AAVS1* (Figure 2B).

Multiple genes can be edited by expressing a single CRISPR-Cas12a crRNA array together with Cas12a protein, taking advantage of its dual DNase and RNase activity (Zetsche et al., 2017). To further understand the potential of multiple-target editing using this system, we then targeted four genes (*AAVS1*, *DYNLT1*, *EMX1*, *DNMT1*) using a crRNA array and inserted mKate into *DYNLT1* locus and GFP into *AAVS1* locus at the same time. Focusing on fluorescent tag knock-in editing, we tested four different array designs with four guides in distinct orders, and achieved successful simultaneous HDR editing of both mKate and GFP with an approximate 2–4.5-fold increase in efficiency using Cas12a-REDIT (Figure 2C). While we maintained the guide sequence across the designs, we observed different editing efficiencies (Figure 2C). This is likely due to the varying processing efficiencies of crRNA array by Cas12a protein. This processing could be influenced by multiple factors. On one hand, the positioning of the crRNA within an array could influence the processing efficiencies, thus leading to variable expression levels of individual guide depending on the position, explaining the results we observed (Campa et al., 2019). On the other hand, recent work (Magnusson et al., 2021) also reported that the guide sequence could also influence the processing efficiencies of Cas12a crRNA arrays, which could also contribute to the differences in editing outcomes when we change the position of crRNAs within an array.

After validating the multi-target editing of the Cas12a-REDIT method, we additionally assayed the off-target effect of our design by profiling the editing events on potential off-target sites (OTS). We examined two genes, *EMX1* and *VEGFA*, and OTS were predicted using http://crispor.org. Based on the score ranking, five *EMX1* OTS and six *VEGFA* OTS were selected (sequences listed in Supplementary Table), and editing efficiencies on these OTS were determined by next-generation sequencing. For both *EMX1* and *VEGFA*, our system exhibited no markable increase in the off-target editing (Figures 2D,E).

In summary, by employing the SSAP activity of phage RecT, we engineered a Cas12a-based system that can increase the efficiency of kilo-base scale HDR editing without compromising its specificity, and such improvements could also be achieved in multiplex genome editing.

In the longer term, additional method development could enhance the utility of this multiplex genome knock-in system. Specifically, many emerging areas of gene-editing research will have significant synergy with our work: protein engineering to define minimal-functional Cas12a-RecT editor including to optimize its efficiency; more powerful delivery systems including for the repair templates, which is very challenging for *in vivo* application of this multiplex editor. These additional advancements will further enhance our approach and help to achieve high-efficiency, multi-target long-sequence editing in human cells.

## Data Availability

The datasets presented in this study can be found in online repositories. The names of the repository/repositories and accession number(s) can be found below: NCBI BioProject PRJNA735023.
