# Supplementary material for: CRISPR-Cas12a System With Synergistic Phage Recombination Proteins for Multiplex Precision Editing in Human Cells
Source: Front Cell Dev Biol. 2022 Jun 14;9:719705. doi: 10.3389/fcell.2021.719705 (PMC9237396; doi:10.3389/fcell.2021.719705)
Supplement: Supplementary file 1 [file Table1.docx]

**Supplementary table**

| Primer name | Usage | Genomic Target | Primer sequence (NGS adapters used for amplification are highlighted in red) |
| --- | --- | --- | --- |
| EMX1-NGS-F | NGS | *EMX1* | 5’-CCATCTCATCCCTGCGTGTCTCC CTCAGTCTTCCCATCAGGCTC-3’ |
| EMX1-NGS-R | NGS | *EMX1* | 5’-CCTCTCTATGGGCAGTCGGTGATG AACTGGAGAAGCCACCAGAGT-3’ |
| EMX1-OT-1 | Off-target |  | 5’-TGGTTGCCCACCATAGGAATCAT-3’ |
| EMX1-OT-2 | Off-target |  | 5’-TGGCTGCCCTCACTACTCATTAG-3’ |
| EMX1-OT-3 | Off-target |  | 5’-TGGCTGGGCTCCCTAGTCATCGG-3’ |
| EMX1-OT-4 | Off-target |  | 5’-TGGTTCCCCACCCAAGGCATATA-3’ |
| EMX1-OT-5 | Off-target |  | 5’-TGTTTTCCCACCCTAGTGACTTT-3’ |
| VEGFA-OT-1 | Off-target |  | 5’-CTTGGAATTTTGAAGGGGGCTGA-3’ |
| VEGFA-OT-2 | Off-target |  | 5’-CTAAGAAGATTGAAGGGGGTGAA-3’ |
| VEGFA-OT-3 | Off-target |  | 5’-CTGGGATTATTGATGGGGGCGGT-3’ |
| VEGFA-OT-4 | Off-target |  | 5’-CTGAGAATATTGAAGGGCACAGA-3’ |
| VEGFA-OT-5 | Off-target |  | 5’-CAGGGAATATTAAAGGGGACAGA-3’ |
| VEGFA-OT-6 | Off-target |  | 5’-ATAGGAAAACTGAAGGAGGCTGG-3’ |
| DYNLT1-PCR-100bp-F | PCR template | *DYNLT1* | 5’-TGCCGTAAATGCTGCTCTCT-3’ |
| DYNLT1-PCR-200bp-F | PCR template | *DYNLT1* | 5’-AGACTTGCCAAGGTTCTTTGTG-3’ |
| DYNLT1-PCR-400bp-F | PCR template | *DYNLT1* | 5’-AGTGACCTGTGTAATTATGCAGAAG-3’ |
| DYNLT1-PCR-100bp-R | PCR template | *DYNLT1* | 5’-TGAAAGTGCCACAAAACAAAGAGA-3’ |
| DYNLT1-PCR-200bp-R | PCR template | *DYNLT1* | 5’-AAGACAAGTGGCAACGCAG-3’ |
| DYNLT1-PCR-400bp-R | PCR template | *DYNLT1* | 5’-CGTTTATGATACTATGCAGACTATGAAGAAC-3’ |
| AAVS1-PCR-100bp-F | PCR template | *AAVS1* | 5’-ACCTCCTGTTAGGCAGATTC-3’ |
| AAVS1-PCR-200bp-F | PCR template | *AAVS1* | 5’-TCCACCCCACAGTGGGG-3’ |
| AAVS1-PCR-400bp-F | PCR template | *AAVS1* | 5’-CTCTGACCTGCATTCTCTCCCC-3’ |
| AAVS1-PCR-100bp-R | PCR template | *AAVS1* | 5’-TTCTGGGAGAGGGTAGCG-3’ |
| AAVS1-PCR-200bp-R | PCR template | *AAVS1* | 5’-TTTCCAAACTGCTTCTCCTCTTGG-3’ |
| AAVS1-PCR-400bp-R | PCR template | *AAVS1* | 5’-TGAACACCTAGGACGCACCATT-3’ |
| DYNLT1-crRNA | Guide RNA | *DYNLT1* | 5’-ACCTGCAGTCCAGCCTATGGCCT-3’ |
| AAVS1-crRNA | Guide RNA | *AAVS1* | 5’-CTGATGACGCGGATCAACGCTGC-3’ |
| EMX1-crRNA | Guide RNA | *EMX1* | 5’-TGGTTGCCCACCCTAGTCATTGG-3’ |
| VEGFA-crRNA | Guide RNA | *VEGFA* | 5’-CTAGGAATATTGAAGGGGGCAGG-3’ |
|  |  |  |  |
