# Supplementary material for: CRISPR-Cas12a System With Synergistic Phage Recombination Proteins for Multiplex Precision Editing in Human Cells
Source: Front Cell Dev Biol. 2022 Jun 14;9:719705. doi: 10.3389/fcell.2021.719705 (PMC9237396; doi:10.3389/fcell.2021.719705)
Supplement: Supplementary file 2 [file DataSheet1.docx]

Supplementary Text

Step-by-step gene-editing protocol using Cas12a-REDIT

A. Design of guideRNA sequences at target genomic loci

We could design the guideRNA targeted specific genimoc locus in Benchling (<https://www.benchling.com/>). Briefly, based on the Cas9 enzyme used, target sequence (usually 20~23-bp) near the knock-in or editing sites can be selected next to the protospacer adjacent motif (PAM). For Cas12a use “TTTN”. Two DNA oligos could be ordered based on selected guides, with golden gate cloning overhangs, as shown below (please note the AGAT and AAAA are the overhang that is needed for cloning into a Cas12a crRNA array backbone vector, this vector should have a Cas12a direct-repeat already in the vector after U6 promoter).

**5’ –AGAT**NNNNNNNNNNNNNNNNNNNNNNN **–3’**

**3’ –**NNNNNNNNNNNNNNNNNNNNNNN**AAAA –5’**

N denotes the guide sequences. Standard desalting oligos are sufficient for this cloning. The two oligos above will be annealed to form the insert fragments in the next step.

B. Annealing of two DNA oligos for each guideRNA target. Perform phosphorylation and annealing of each pair of oligos via reaction setup below.

| oligo1 Top (100 uM) | 1 ul |
| --- | --- |
| oligo2 Bottom (100 uM) | 1 ul |
| 10X T4 ligation Buffer(NEB) | 1 ul |
| ddH_2_O | 6.5 ul |
| T4 PNK (NEB) | 0.5 ul |
| Total | 10 ul |

Anneal in a thermocycler using the following parameters:

37C 35 min

95C 5 min and then ramp down to 25C at 5C/min or take the tubes out and put on the bench to let them recover to room temperature.

C. Golden Gate Cloning of annealed oligos into Cas12a-guide RNA backbone plasmid

For Cas12a-REDIT test, one guide RNA is needed and the backbone vectors for the cloning will bear Esp3I cloning sites matching the annealed oligos from Step B.

| **Item** | **Volume** | **Note** |
| --- | --- | --- |
| Water | 4.3 ul |  |
| Cutsmart Buffer | 0.8 ul | 10x |
| T4 ligase | 0.2 ul |  |
| Esp3I | 0.4 ul |  |
| ATP (25mM) | 0.3 ul | ~ final 1mM |
| plasmid/vector | 1 ul | ~ 50ng total plasmid |
| Annealed Oligo (1:10 diluted) | 1 ul | diluted 10ul into 100ul |
| Total | 8 ul |  |

This protocol uses a minimal amount of enzyme and could be scaled up as needed. After set up the golden gate reaction (on ice), immediately move the reaction into Thermocycler and perform the golden gate reaction using the following parameters:

37C 5 min

16C 5 min

Cycle for ~25 cycles

65C 5 min

4C hold

After the reaction, perform bacterial transformation as per standard protocol of the competent cells used in the lab immediately.

12~18 hours after transformation, pick colonies and culture in LB media with Carbenicillin antibiotic for another 12~14 hours at 37C.

Do miniprep using QIAprep Spin Miniprep Kit and confirm guide RNA sequence using Sanger sequencing. Could perform the following step if the sequences are correct.

D. Preparation of HDR templates

Please refer to Supplementary Table for primers used in the. We recommend using a dsDNA template with at least 200bp of homology arms on each end of the insertion sequences. We suggest cloning the template into simple plasmids such as pUC19, then, restriction digestion of plasmids or standard PCR (using primers such as listed in the Supplementary table) could be employed for generating large amounts of dsDNA templates.

E. Perform gene-editing via delivery of Cas12a-SSAP plasmids and template DNA

With previous steps, the 3 components of Cas12a-SSAP editing method are ready for experiments: the guideRNA/Cas12a plasmid (cloned in step A-C), the template DNA (from step D), and the SSAP plasmid (scFV-RecT, can be obtained from Addgene). For delivery into cells *in vitro*, routine transfection or electroporation could be performed following the recommended conditions by the reagent or equipment manufacturer and selected based on the cell types. For HEK293T cells as an example, a typical transfection condition is described below:

1. 12 hours before transfection, 3E4 HEK293T cells seeded on each well of 96-well plate, the cell density should be around 70% on the next day at the time of transfection. For Hela and HepG2 cells, seed 48-well plates at the 5E4 cells/well and 3E4 cells/well density respectively.
2. For lipofectamine 3000 as the transfection reagent, use a total of 250 ng DNA + 0.4 ul Lip3000 reagents (ea.) for HEK293T cells and perform the reagent set up using 10 ul of Opti-MEM per well, as in the manufacturer's protocol. For Hela and HepG2 cells, we use 400ng of togal DNA with 1.0 ul Lip3000 reagents for each well.
3. Transfection material:

For HEK293T cells: guideRNA/Cas12a plasmid, 160ng; scFV-RecT or GFP control plasmid, 60ng; Template DNA, up to 30ng.

For Hela and HepG2 cells: guideRNA/Cas12a plasmid, 250ng; scFV-RecT or GFP control plasmid, 100ng; Template DNA, up to 50ng.

1. Mix plasmids with template DNA and perform transfection according to the manufacturer's protocol.
2. 12-24 hours after transfection, if applicable could switch to fresh media.
3. 72 hours post transfection, cells could be harvested or proceed to downstream experiments or analysis as needed.
